# In vivo human molecular neuroimaging of dopaminergic vulnerability along the Alzheimer’s disease phases

**DOI:** 10.1186/s13195-021-00925-1

**Published:** 2021-11-12

**Authors:** Arianna Sala, Silvia Paola Caminiti, Luca Presotto, Andrea Pilotto, Claudio Liguori, Agostino Chiaravalloti, Valentina Garibotto, Giovanni Battista Frisoni, Marcello D’Amelio, Barbara Paghera, Orazio Schillaci, Nicola Mercuri, Alessandro Padovani, Daniela Perani

**Affiliations:** 1 Vita-Salute San Raffaele University, Via Olgettina 60, Milan, 20132 Italy; 2 In Vivo Human Molecular and Structural Neuroimaging Unit, Division of Neuroscience, San Raffaele Scientific Institute, 20132 Milan, Italy; 3 Nuclear Medicine Unit, San Raffaele Hospital, 20132 Milan, Italy; 4 Neurology Unit, Department of Clinical and Experimental Sciences, University of Brescia, 25121 Brescia, Italy; 5 Parkinson’s Disease Rehabilitation Centre, FERB ONLUS – S. Isidoro Hospital, 24069 Trescore Balneario, Italy; 6 Division of Neurology, Department of Systems Medicine, University of Rome “Tor Vergata”, 00133 Rome, Italy; 7 Department of Biomedicine and Prevention, University of Rome “Tor Vergata”, 00133 Rome, Italy; 8 IRCCS Neuromed, 86077 Pozzilli, Italy; 9 Division of Nuclear Medicine and Molecular Imaging, Diagnostic Department, University Hospitals of Geneva, and NIMTLab, Faculty of Medicine, Geneva University, 1205 Geneva, Switzerland; 10 Memory Clinic and LANVIE-Laboratory of Neuroimaging of Aging, University Hospitals and University of Geneva, 1205 Geneva, Switzerland; 11 Department of Experimental Neurosciences, IRCCS Santa Lucia Foundation, 00179 Rome, Italy; 12 Unit of Molecular Neurosciences, Department of Medicine, University Campus-Biomedico, 00128 Rome, Italy; 13 Nuclear Medicine Unit, Spedali Civili Brescia, 25123 Brescia, Italy

**Keywords:** Biomarker, Dopamine, Molecular connectivity, Substantia nigra, Ventral tegmental area

## Abstract

**Background:**

Preclinical and pathology evidence suggests an involvement of brain dopamine (DA) circuitry in Alzheimer’s disease (AD). We in vivo investigated if, when, and in which target regions [123I]FP-CIT-SPECT regional binding and molecular connectivity are damaged along the AD course.

**Methods:**

We retrospectively selected 16 amyloid-positive subjects with mild cognitive impairment due to AD (AD-MCI), 22 amyloid-positive patients with probable AD dementia (AD-D), and 74 healthy controls, all with available [123I]FP-CIT-SPECT imaging. We tested whether nigrostriatal vs. mesocorticolimbic dopaminergic targets present binding potential loss, via MANCOVA, and alterations in molecular connectivity, via partial correlation analysis. Results were deemed significant at *p* < 0.05, after Bonferroni correction for multiple comparisons.

**Results:**

We found significant reductions of [123I]FP-CIT binding in both AD-MCI and AD-D compared to controls. Binding reductions were prominent in the major targets of the ventrotegmental-mesocorticolimbic pathway, namely the ventral striatum and the hippocampus, in both clinical groups, and in the cingulate gyrus, in patients with dementia only. Within the nigrostriatal projections, only the dorsal caudate nucleus showed reduced [123I]FP-CIT binding, in both groups. Molecular connectivity assessment revealed a widespread loss of inter-connections among subcortical and cortical targets of the mesocorticolimbic network only (poor overlap with the control group as expressed by a Dice coefficient ≤ 0.25) and no alterations of the nigrostriatal network (high overlap with controls, Dice coefficient = 1).

**Conclusion:**

Local- and system-level alterations of the mesocorticolimbic dopaminergic circuitry characterize AD, already in prodromal disease phases. These results might foster new therapeutic strategies for AD. The clinical correlates of these findings deserve to be carefully considered within the emergence of both neuropsychiatric symptoms and cognitive deficits.

**Supplementary Information:**

The online version contains supplementary material available at 10.1186/s13195-021-00925-1.

## Background

The role of dopaminergic neurotransmission circuits in the pathophysiology of Alzheimer’s disease (AD) is currently debated. Evidence of dopaminergic dysfunction in AD traces back to a pivotal binding study, reporting decreased bindings of [3H]Spiroperidol in the caudate nucleus of six brains of patients with autopsy-confirmed AD [1]. Several post-mortem studies subsequently showed alterations in the substantia nigra (SN) [2–6] and ventral tegmental area (VTA) [3] and both pre- [7–11] and post-synaptic [5, 12–18] neurotransmission alterations in several dopaminergic targets [5, 7, 8, 10, 11, 14–16, 18]. These pathology results were complemented, more recently, by more limited neuroimaging evidence, reporting alterations in the striatal [19–23] and hippocampal [24] dopaminergic function limitedly to patients with overt AD dementia (AD-D).

Several questions remained unanswered. First, it is unclear whether dopaminergic dysfunction represents an early vs. late occurrence along the AD course. Second, it is unknown whether the nigrostriatal and the mesocorticolimbic dopaminergic pathways are differently affected in AD and whether specific targets are more vulnerable than others. Lack of comprehensive information about dopaminergic deficits in AD hinders the discovery and application of neurotransmission-targeting therapeutic strategies along the AD course.

In the present study, we aimed to assess (i) the extent of pre-synaptic dopaminergic dysfunction in AD, (ii) when it takes place along the disease course, and (iii) which specific targets belonging to the nigrostriatal and mesocorticolimbic dopaminergic pathways are affected. We used complementary analytical strategies, namely the evaluation of *regional* [123I]FP-CIT binding in each dopaminergic pathway and the assessment of their *molecular connectivity* alterations [25].

## Methods

### Study design

Participants with AD-D [26] and with mild cognitive impairment due to AD [27] (AD-MCI) and healthy controls (HC) were retrospectively collected from three clinical centers: University of Brescia (Brescia, Italy; 4 AD-D, 7 AD-MCI, 43 HC, acquisition period 2013–2018), Geneve University Hospital (Geneve, Switzerland; 11 AD-D, 2 AD-MCI, 31 HC, acquisition period 2008–2017), and University Hospital of Rome Tor Vergata (Rome, Italy; 7 AD-D, 7 AD-MCI, acquisition period 2012–2013).

All patients underwent structural imaging (MRI or CT scan) and standardized neurological examinations. The following conditions were excluded: (1) atypical parkinsonism/dementia; (2) frontotemporal dementia; (3) prominent cortical or subcortical infarcts; (4) other neurologic or psychiatric diseases or medical conditions potentially associated with cognitive deficits; (5) history of drug or alcohol abuse, use of antipsychotics; and (6) use of antidepressant or serotonergic drugs which can interfere with [123I]FP-CIT SPECT acquisition [28].

We included only subjects belonging to the AD continuum (i.e., amyloid-positive), in agreement with the recently proposed NIA-AA research framework [29]. Amyloid positivity was established based on CSF-Aβ42 in *N* = 25 cases and on amyloid-PET in the remaining *N* = 13 cases, in accordance with the CSF cut-offs used in each clinical center or based on amyloid-PET positivity.

Patients underwent [123I]FP-CIT SPECT imaging for research purposes (University of Brescia) or for clinical purposes (Geneve University Hospital; University Hospital of Rome Tor Vergata), i.e., to exclude a dementia with Lewy bodies (DLB) diagnosis. In all the included clinical cases, [123I]FP-CIT-SPECT scans were rated as negative according to a predefined ranking scale [28].

**Fig. 1:**
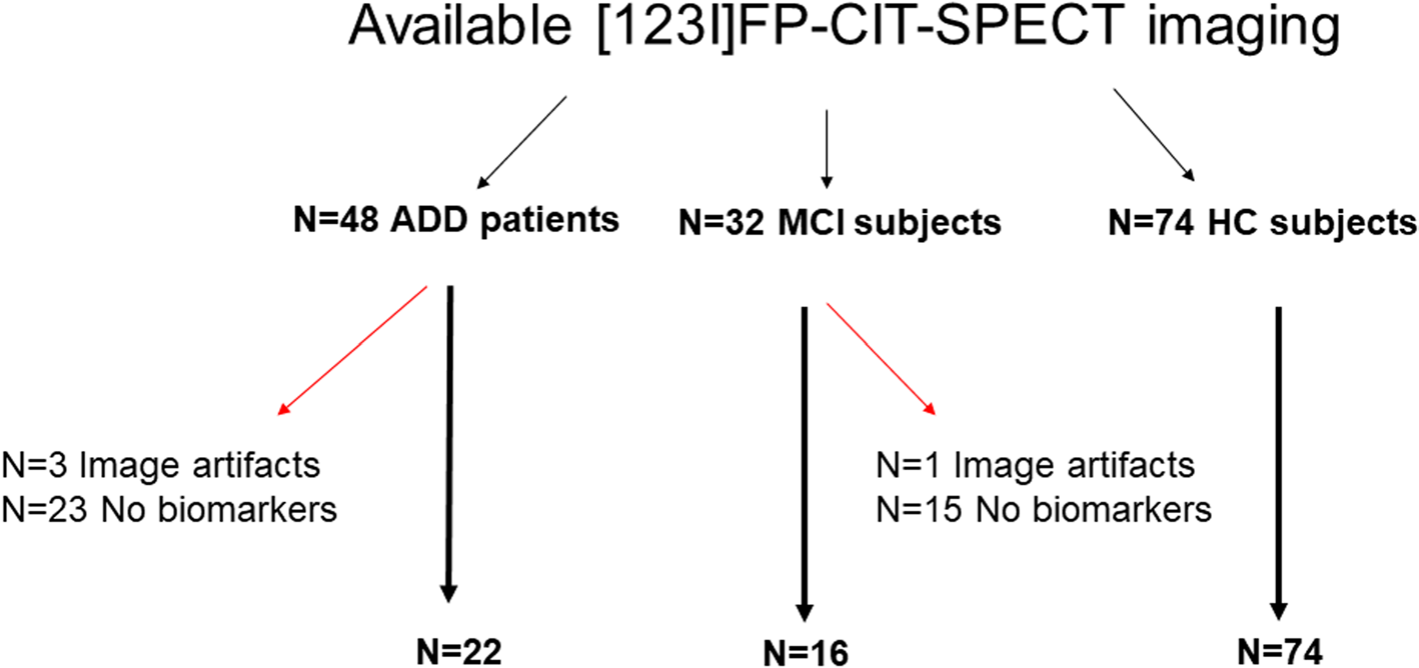
Flow diagram showing the number of subjects/patients who underwent [123I]FP-CIT SPECT imaging and were initially screened for the present study. Red arrows indicate those individuals that were excluded

A group of 74 healthy volunteers was included in the study as HC (Table 1). They presented a negative medical history for neurological disease and were not taking psychoactive medication. All subjects presented with a confirmed clinical diagnosis of isolated action or rest tremor syndromes over a 4-year follow-up and normal [123I]FP-CIT binding [30, 31]. See Fig. 1 for a flow diagram depicting the included/excluded individuals.

**Table 1:** Descriptive demographic and clinical features of the study groups

	AD-D	AD-MCI	HC	Test value; ***p***
***N***	22	16	74	–
**Sex,** ***N*** **(M/F)**	10/12	11/5	31/43	–
**Age, mean ± SD**	72.18 ± 6.27	71.38 ± 10.53	67.15 ± 13.87	*F* = 1.84; *p* = 0.16
MMSE, mean ± SD*	17.92 ± 6.70	26.88 ±1.20	–	*T* = 4.75; *p* < 0.001
CDR, mean ± SD*	1.84 ± 0.77	0.50 ± 0.00	–	*T* = −7.65; *p* < 0.001

*Abbreviations*: *AD-D* Alzheimer’s disease dementia, *CDR* Clinical Dementia Rating, *HC* healthy controls, *AD-MCI* MCI due to Alzheimer’s disease, *MMSE* Mini-Mental State Examination

*Neither MMSE nor CDR was available in 3 AD-D patients, who were tested by means of the Montreal Cognitive Assessment test (total MoCA score = 11; 12; 19, respectively)

Demographic differences between groups were evaluated by means of ANOVA and chi-squared tests.

### [123I]FP-CIT SPECT

Intravenous administration of 110–185 MBq of [123I]FP-CIT was performed 30 min after thyroid blockade (800 mg of KClO4) in all subjects. Brain SPECT acquisitions were performed 3 to 4 h after injection with the following protocols: (i) at the University of Brescia and (ii) at the University of Rome Tor Vergata using a dual-head gamma camera equipped with a LEHR parallel hole collimator (Discovery 630, General Electric, Milwaukee, WI) accepting events in a 159-KeV photopeak ±10% energy window. Data were reconstructed by filtered back projection, with Butterworth 3-dimensional (3D) post-filter (order 10.0; cut-off 0.50 cycle/cm) and corrected for attenuation (Chang’s method coefficient 0.15 cm^−1^)); (iii) at Geneva University Hospitals acquisitions were performed based on a three head camera (Toshiba GCA-9300A) with fan beam collimators using a triple energy window for scatter correction. Data were reconstructed using filtered back projection with a Shepp-Logan filter at the Nyquist frequency and corrected for attenuation (Chang’s method, uniform attenuation coefficient of 0.12/cm^−1^, accounting for the scatter corrections). A single reconstruction protocol was used for centers 1 and 2, in order to produce comparable data. For the Geneva center, this was not possible as the gamma camera used had different (and improved) physical proprieties. The reconstruction method was introduced as a covariate in the following analysis. As this center provides both patients and control subjects, this is not expected to affect the results of the analysis.

Image pre-processing and quantification were centralized at San Raffaele Hospital (Milan). Molecular images were normalized based on a high-resolution [18F]DOPA template (http://www.nitrc.org/projects/spmtemplates) [32]. Patients’ images were spatially normalized using Statistical Parametric Mapping 12 (SPM12, http://www.fil.ion.ucl.ac.uk/spm/software/spm12). Parametric images were generated for each subject using the Image Calculator (ImCalc) function in SPM12. Specifically, specific binding ratios (SBRs) were calculated using the following formula:

$$\mathrm{SBR}=\frac{{\mathrm{voxel}}_{\mathrm{i}}}{\mathrm{occipital}\ \mathrm{lobe}}-1$$

where [123I]FP-CIT binding counts of three occipital lobe slices was used as the reference region [28].

For the dopaminergic system analysis, we considered regions of interest (ROIs) pertaining to the nigrostriatal and the mesocorticolimbic dopaminergic pathways, as described elsewhere (cfr [33].). The VTA and SN were not included in the analysis, due to the limited spatial resolution of SPECT imaging. The mesocorticolimbic targets consisted of the ventral striatum, anterior and middle cingulate cortices, and ventral and medial frontal areas, as well as the amygdala and parahippocampal cortex; the nigrostriatal targets consisted of the dorsal caudate nucleus and dorsal putamen, frontal premotor, motor, executive dorsolateral frontal regions, and somatosensory cortex. We limited our analyses exclusively to regions belonging to the nigrostriatal and mesocorticolimbic structures and pathways, very rich in dopamine transporters [34].

Each ROI mask was convolved with an 8-mm FWHM Gaussian kernel in order to minimize the partial volume effect.

### Statistical analysis

Mean [123I]FP-CIT SBR within each ROI was extracted from each normalized parametric image. The subcortical/cortical dopaminergic targets that showed significant tracer binding, as compared to the reference region, were selected for further analysis (one-sample *T*-test, *p* < 0.05, Bonferroni-corrected for multiple comparisons). This procedure resulted in a pool of *N* = 10 ROIs belonging to the mesocorticolimbic dopaminergic pathway (L/R ventral striatum, L/R hippocampus, L/R amygdala, L/R anterior cingulate cortex, L/R middle cingulate cortex) and *N* = 4 ROIs belonging to the nigrostriatal dopaminergic pathway (L/R dorsal caudate nucleus, L/R dorsal putamen).

#### Univariate analysis

Regional differences in [123I]FP-CIT SBR between participants with AD-D, AD-MCI, and HC were tested via MANCOVA. The mean SBRs obtained from each ROI were included as dependent variables; age, gender, and reconstruction method were included as nuisance covariates. Results of the MANCOVA were deemed significant at *p* < 0.05, following Bonferroni correction for multiple comparisons (*N* = 14). Pairwise post hoc analyses were subsequently run on significant MANCOVA results, setting the significance threshold at *p* < 0.05, Bonferroni-corrected for multiple comparisons (*N* = 3). Statistical analyses were performed using Statistical Package for the Social Sciences (SPSS19). In order to further characterize the results of the regional analysis, a complementary voxel-based analysis was run, assessing the voxel-wise distribution of [123I]FP-CIT SBR differences obtained in the previous analytical step. [123I]FP-CIT SBR parametric images of AD-D vs. HC and AD-MCI vs. HC were compared by means of a two-sample *T*-test in SPM12, running in MATLAB (Mathworks Inc., Sherborn, Mass., USA). Resulting T-maps were converted into Cohen’s *d* effect size maps by the following formula:

$$D=\frac{2T}{\sqrt{df}}$$

#### Multivariate analysis

Molecular connectivity was estimated following the principle that neurotransmitter release is correlated among territories receiving dopaminergic projections from the same afferents (cf. [25]). Investigation of these patterns of molecular connectivity has provided, in vivo, results consistent with the known biochemical architecture of the dopaminergic system in normal subjects [25]. Assessment of molecular connectivity between targets of each dopaminergic pathway (nigrostriatal; mesocorticolimbic) was performed via partial correlation analysis (cfr [25]). This procedure resulted in the estimation of the molecular structure of each dopaminergic pathway in each group. In order to estimate the molecular connectivity strength between dopaminergic nodes, a partial correlation matrix was computed for each clinical group by means of MATLAB’s *parrcorr* function. Partial correlations allow to investigate the relationship between two regions, while factoring out the contributions of all other regions (cf. [25]). Gender, age, and reconstruction method were included as nuisance covariates. The resulting dopaminergic networks were formed by nodes, represented by the aforementioned ROIs, and by edges, represented by the estimated partial correlation coefficients. Partial correlation coefficients were deemed significant at *p* < 0.01, uncorrected for multiple comparisons, and at *p* < 0.05, Bonferroni-corrected for multiple comparisons. In order to provide an estimate of the overall degree of integrity of each molecular dopaminergic network, we computed an index of similarity between clinical groups and HC, by means of the Dice coefficient.

## Results

We included 22 patients diagnosed with probable AD-D; 16 subjects with AD-MCI, in accordance with current clinical/research criteria [26, 27]; and 74 age-matched HC, all with available [123I]FP-CIT SPECT imaging. AD-D and AD-MCI subjects were comparable for age and sex distribution, differing for MMSE and CDR (*p* < 0.001) (Table 1).

### Univariate analysis

**Fig. 2:**
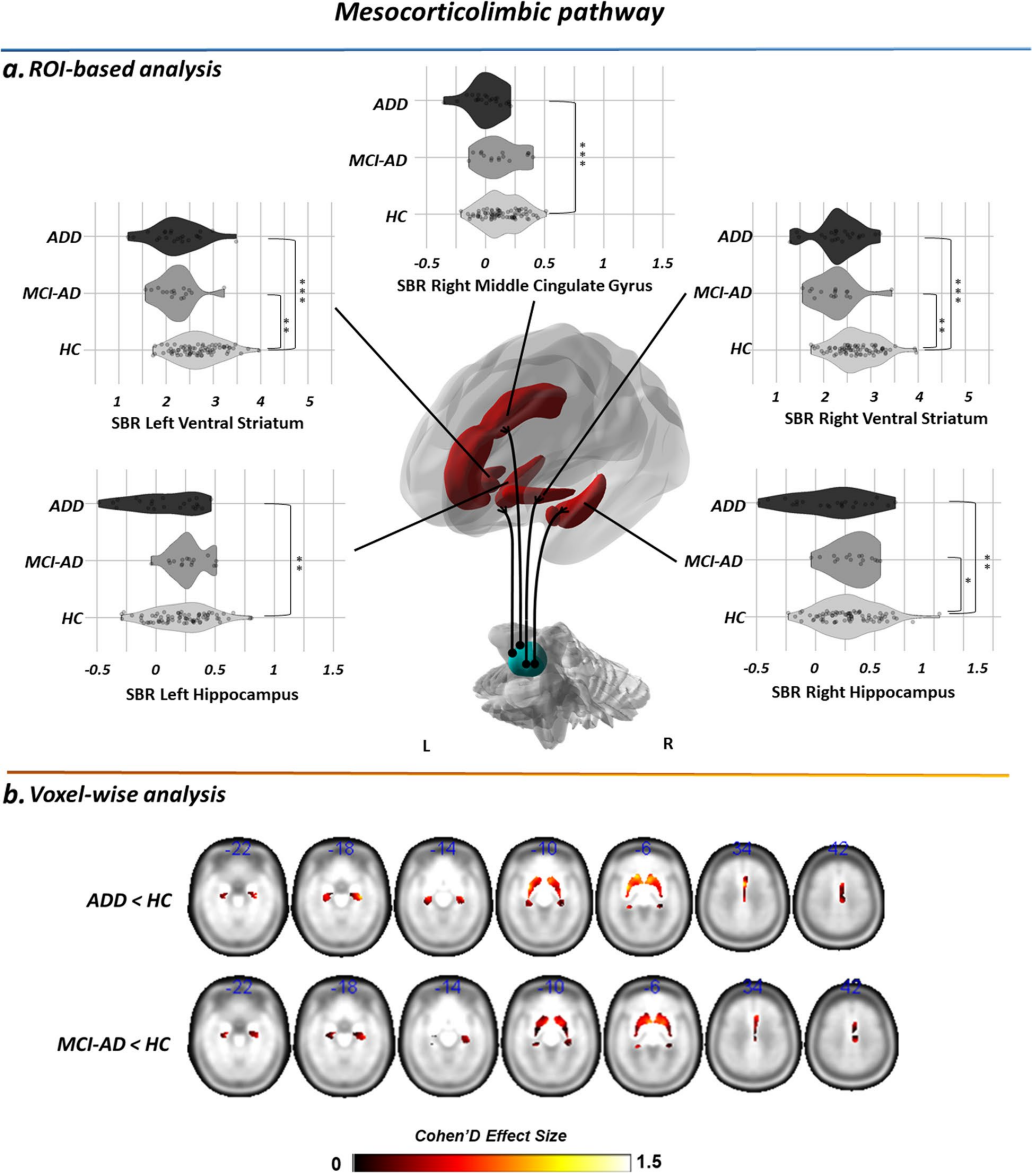
Regions of interest within the mesocorticolimbic pathway showing significant decreases in pre-synaptic dopaminergic activity in AD. **A** Violin plots represent the distribution of SBR in ROIs with significantly decreased DAT density (*p* < 0.05, Bonferroni-corrected for multiple comparisons). Asterisks denote post hoc comparisons, Bonferroni correction for multiple comparisons, at *p* < 0.05 (*), *p* < 0.01 (**), and *p* < 0.001 (***). Brain renderings were obtained from the BrainNet Viewer toolbox [35]. **B** Brain renderings showing the distribution of voxel-wise differences in [123I]FP-CIT BPs in each clinical group, resulting from statistical comparison with HC. The magnitude of the difference is reported by means of Cohen’s *d* effect size. Only ROIs showing significantly decreased [123I]FP-CIT BP in association with Alzheimer’s disease are shown. Abbreviations: AD-D, Alzheimer’s disease dementia; AD-MCI, mild cognitive impairment due to Alzheimer’s disease; HC, healthy controls

**Fig. 3:**
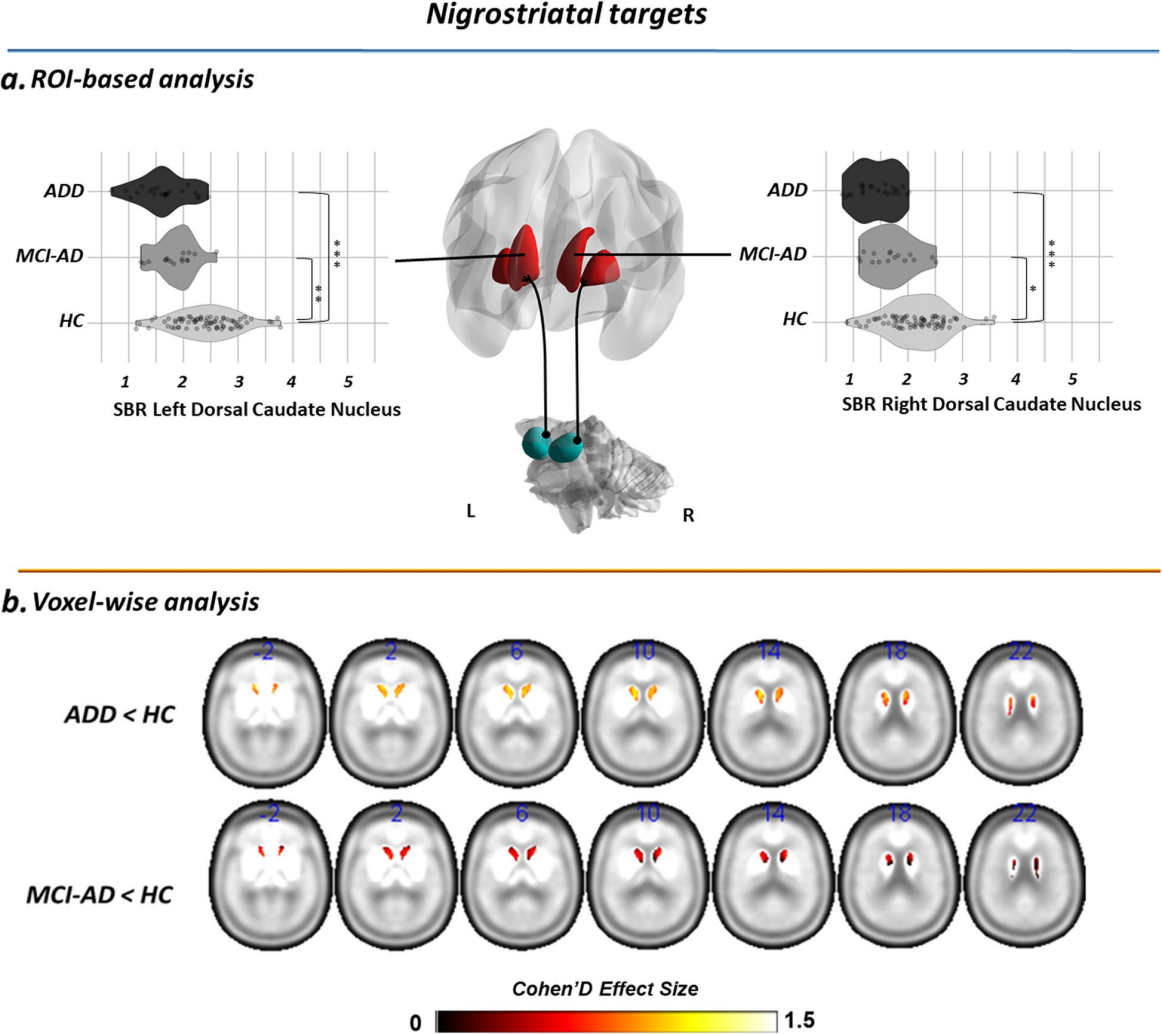
Regions of interest within the nigrostriatal pathway showing significant decreases in pre-synaptic dopaminergic activity in AD. **A** Violin plots represent the distribution of SBR in ROIs with significantly decreased DAT activity (*p* < 0.05, Bonferroni-corrected for multiple comparisons). Asterisks denote post hoc comparisons, Bonferroni correction for multiple comparisons, at *p* < 0.05 (*), *p* < 0.01 (**), and *p* < 0.001 (***). Brain renderings were obtained from the BrainNet Viewer toolbox [35]. **B** Brain renderings show the distribution of voxel-wise differences in [123I]FP-CIT BP in each clinical group, resulting from statistical comparison with healthy controls. The magnitude of the difference is reported by means of Cohen’s *d* effect size. Only ROIs showing significantly decreased [123I]FP-CIT BP in association with Alzheimer’s disease are shown. Abbreviations: AD-D, Alzheimer’s disease dementia; AD-MCI, mild cognitive impairment due to Alzheimer’s disease; HC, healthy controls

Results of the MANCOVA, comparing [123I]FP-CIT SBR in the major subcortical/cortical targets of the dopaminergic pathways, among AD-D, AD-MCI, and HC, are shown in Figs. 2A, and 3A and Table A.1. Molecular imaging assessment demonstrated a significant reduction of [123I]FP-CIT SBR in both AD-MCI and AD-D, predominantly in the regions belonging to the mesocorticolimbic dopaminergic pathway.

As for the mesocorticolimbic pathway, AD-D showed a decreased [123I]FP-CIT SBR in the main subcortical and cortical targets of the VTA, namely the ventral striatum and hippocampus, bilaterally, and the right middle cingulate gyrus, compared to control subjects. Similar alterations were reported in AD-MCI compared to HC, with decreased [123I]FP-CIT SBR in the ventral striatum, bilaterally, and the right hippocampus. No significant differences in [123I]FP-CIT SBR were detected between AD-D and AD-MCI in any mesocorticolimbic target (*p* < 0.05, Bonferroni-corrected for multiple comparisons).

As for the nigrostriatal pathway, only the dorsal caudate nucleus showed decreased [123I]FP-CIT SBR, bilaterally, in both the AD-D and AD-MCI compared to HC. No differences in DAT density were detected in any other nigrostriatal target.

Voxel-wise analysis, independently assessing differences between each clinical group vs. HC, showed that the strongest alterations in DAT density (Cohen’s *d* > 1) were localized in the ventral striatum (peak MNI coordinates: *x* = −6; *y* = 8; *z* = −6 [AD-MCI]; *x* = 8; *y* = 14; *z* = −4 [AD-D]) for the mesocorticolimbic pathway and in the caudate head (peak MNI coordinates: *x* = −8; *y* = 8; *z* = 0 [AD-MCI]; *x* = −12; *y* = 12; *z* = 12 [AD-D]) for the nigrostriatal pathway (Figs. 2B and 3B).

### Multivariate analysis

**Fig. 4:**
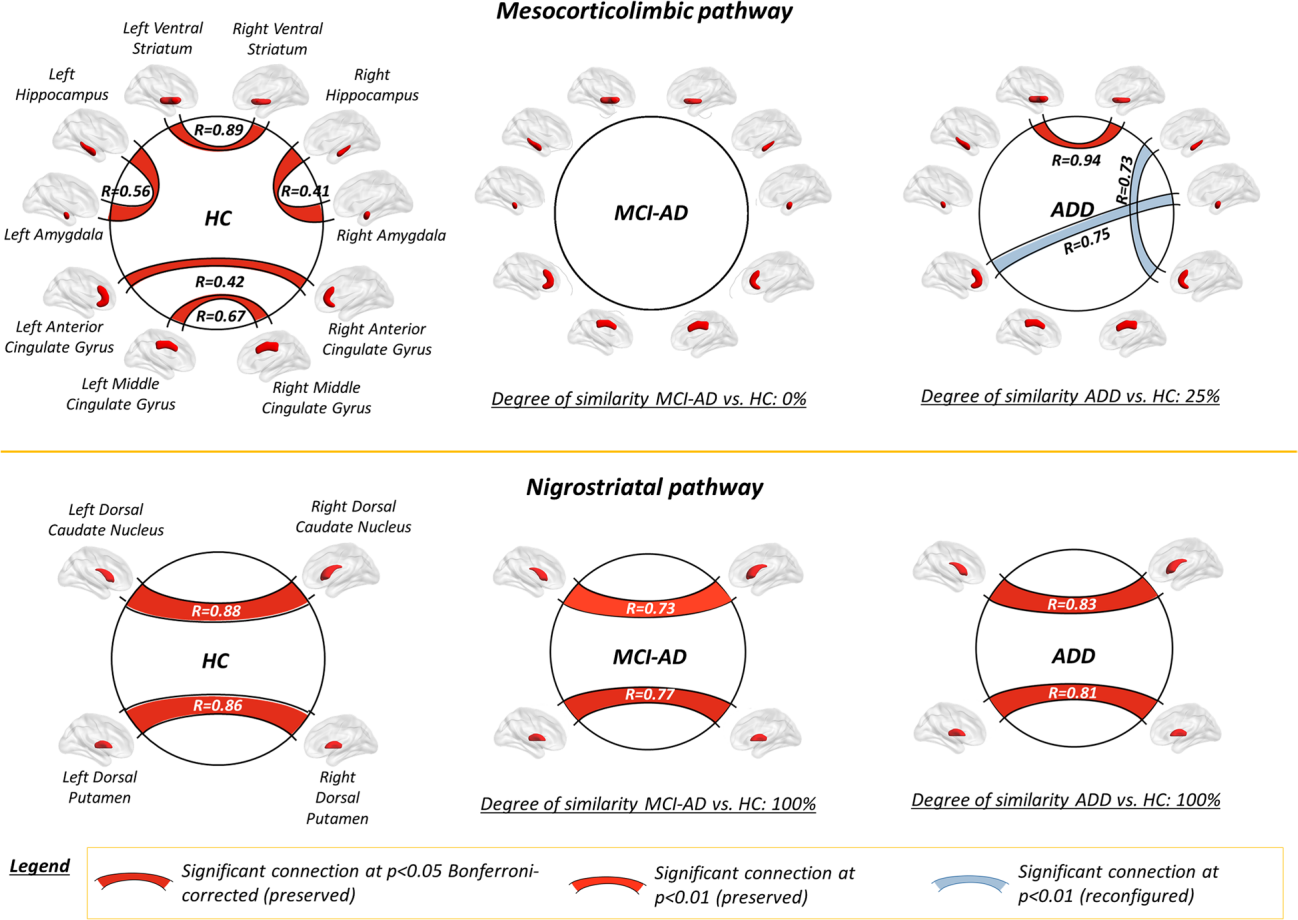
Molecular connectivity of dopaminergic networks in healthy controls and AD. Brain renderings show the molecular structure of the nigrostriatal and mesocorticolimbic dopaminergic networks at *p* < 0.05, Bonferroni-corrected for multiple comparisons (red edges), and *p* < 0.01, uncorrected for multiple comparisons (orange edges). Overlap between molecular connectivity networks in healthy controls and each clinical group is also shown on the right, as computed by means of the Dice coefficient. Brain renderings were obtained from the BrainNet Viewer toolbox [35]. Abbreviations: AD-D, Alzheimer’s disease dementia; AD-MCI, mild cognitive impairment due to Alzheimer’s disease; HC, healthy controls

Results of the multivariate analysis are summarized in Fig. 4. Molecular connectivity assessment demonstrated a widespread loss of inter-connections between subcortical and cortical targets of the mesocorticolimbic pathway, in AD-D and AD-MCI (*p* < 0.01, uncorrected for multiple comparisons; *p* < 0.05, Bonferroni-corrected for multiple comparisons). No connectivity alterations were found within the nigrostriatal pathway, with preserved caudate and putaminal inter-connections in both clinical groups (*p* < 0.01, uncorrected for multiple comparisons). Quantitative assessment of the overall integrity of each dopaminergic pathway, by means of Dice coefficient, confirmed that molecular connectivity was severely altered within the mesocorticolimbic network (Dice coefficient = 0 [AD-MCI]; 0.25 [AD-D], indicating poor to fair similarity): Molecular connectivity appeared to be preserved in AD-D and AD-MCI within the nigrostriatal network (Dice coefficient = 1 [AD-MCI]; 1 [AD-D], indicating excellent overlap) (*p* < 0.01, uncorrected for multiple comparisons).

## Discussion

In vivo research studies on neurotransmission alterations are crucial to provide biological-based evidence of molecular alterations in neurodegenerative diseases, supporting available symptomatic therapeutic strategies and fostering drug discovery. In this study, we provide unique in vivo evidence for specific alterations of the dopaminergic pathways along the AD stages in a well-characterized sample of amyloid-positive individuals with AD-D and AD-MCI. We demonstrated that the dopaminergic projections arising from the VTA are the most vulnerable in AD, with a significant reduction of [123I]FP-CIT SBR in the major mesocorticolimbic targets, since the prodromal disease phases (Fig. 2). We also found extensive alterations of the molecular architecture of the mesocorticolimbic pathway (Fig. 4). The dopaminergic projections arising from the SN were instead spared, with loss of [123I]FP-CIT SBR limited to the head of the caudate nucleus (Fig. 3), and no network alteration in its molecular circuitry (Fig. 4).

The first finding of this study pertains to the presence of significant reductions of [123I]FP-CIT SBR in several targets of the dopaminergic pathways in AD (Figs. 2 and 3). These results are supported by recent results obtained on a validated mouse model of AD [36, 37] and corroborated by previous *post-mortem* [2, 7–11] and in vivo imaging evidence [19, 38], reporting pre-synaptic dopaminergic dysfunction in AD-D. We also provide a new remarkable finding of system-level dopaminergic alterations, with disruption of the pattern of regional connectivity observed in HC (Fig. 4). Abnormal patterns of dopaminergic connectivity were previously reported in neurodegenerative disease characterized by known dopaminergic deficits, such as Parkinson’s disease and DLB, showing prominent alterations within the nigrostriatal system (cf. [25]). The present new in vivo findings demonstrate alterations of the neurotransmission architecture in AD as well, but within the mesocorticolimbic system.

**Fig. 5:**
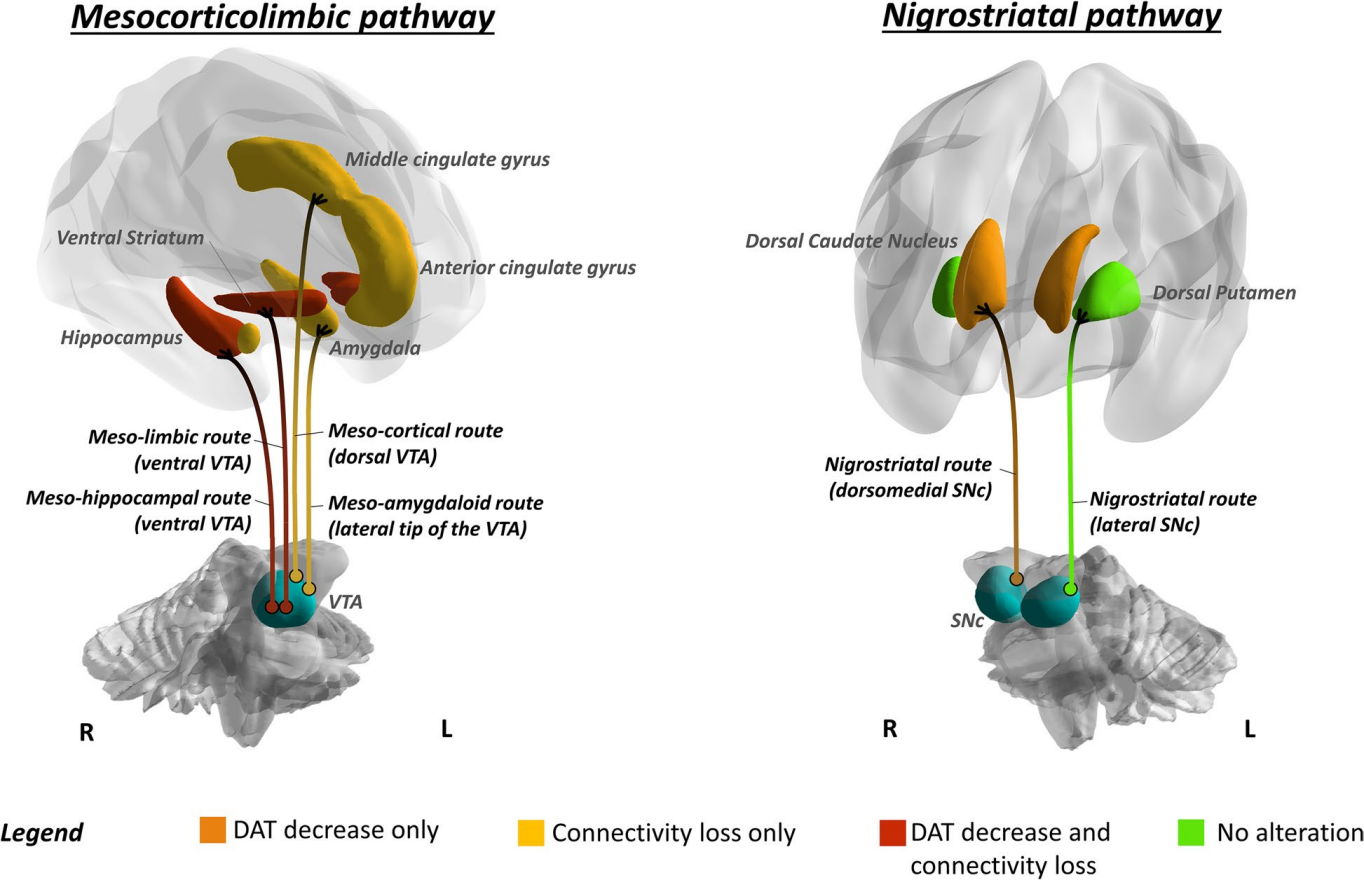
Summary representation of dopaminergic dysfunction in early AD. Brain renderings show dopaminergic targets presenting with decreased DAT activity (orange), loss of molecular connectivity (yellow), or both (red) in prodromal AD. Alterations in these target regions can be deemed indicative of pre-synaptic dopaminergic dysfunction in specific afferents from the ventral and dorsal VTA and from the medial SN, pars compacta. For the purpose of ensuring a clear visualization, dopaminergic projections are represented only for each target region on the ipsilateral side. Brain renderings were obtained from the BrainNet Viewer toolbox [35]. Abbreviations: SNc, substantia nigra pars compacta; VTA, ventral tegmental area

The second main finding of this study pertains to the disease stage in which dopaminergic deficits occur in AD. Available in vivo studies provided evidence for dopaminergic alterations exclusively in advanced disease stages [19–23]. No in vivo studies are available directly assessing dopamine pathophysiology in AD-MCI. Here, we found, already at the stage of MCI, a significant loss of DAT density, at a degree comparable to that observed in patients with dementia, in several dopaminergic targets (Fig. 3). The lack of significant differences in regional DAT density between participants with AD-MCI vs. AD-D suggests that dopaminergic dysfunction is an early event, also plateauing early along the disease course. Subjects with AD-MCI showed extensive alterations in the molecular architecture of the mesocorticolimbic dopaminergic circuitry, with loss of inter-connections at the level of the ventral striatum, amygdala, hippocampus, and anterior and middle cingulate gyri. The widespread derangement of molecular connectivity, exceeding the reported [123I]FP-CIT SBR reductions (Fig. 5), indicates an early dysfunction of the dopaminergic circuitry in AD, possibly contributing to its pathophysiology, also at consistency with previous structural and functional connectivity studies [33, 39, 40].

The third main finding of this study pertains to the different vulnerability of the two main dopaminergic pathways in AD. Previous in vivo and *post-mortem* studies focused mainly on the nigrostriatal dopaminergic pathway, reporting alterations in the SN [2–6] and in its major subcortical targets, i.e., putamen [8, 11, 15, 16, 23, 41] and caudate nucleus [7, 11, 16, 23, 41]. There are however several studies reporting lack of alterations in the aforementioned regions [1, 5, 8–10, 12, 13, 17, 23, 38]. Of note, most of the available evidence reports dopaminergic alterations without partitioning the striatum into its ventral and dorsal components, with few exceptions [21]. Our findings partially support the involvement of the nigrostriatal pathway in AD, with [123I]FP-CIT SBR reductions limited to the caudate nucleus, but no alterations in molecular connectivity (Figs. 3 and 4). The caudate nucleus, part of the “associative-cognitive loop,” receives its major dopaminergic input from the dorsomedial portions of the SN pars compacta [42]. Notably, previous post-mortem evidence reported alterations in pre-synaptic dopaminergic function specifically in the dorsal tier of SN pars compacta in AD, at difference to what is observed in Parkinson’s disease, where the ventral portion of the SN pars compacta (and relative projections) are mainly affected [2]. Altogether, this evidence suggests a certain degree of vulnerability of the nigrostriatal pathway in AD, with a very different topography compared to Parkinson’s disease.

Compared to the nigrostriatal pathway, the mesocorticolimbic pathway has been far less studied in AD. Previous* post-mortem* and in vivo neuroimaging evidence suggests an involvement of the VTA [3] and of its targets, i.e., nucleus accumbens [5, 10, 16], limbic striatum [21], hippocampus [18, 23], amygdala [8, 11, 18], and cingulate gyrus [11], with some studies reporting however negative results [1, 8, 10, 12, 23, 41]. Alterations of the mesocorticolimbic targets have been associated with neuropsychiatric symptoms in AD [21, 33], present in up to 90% of patients with dementia [43]. Depression and apathy are the most frequently reported symptoms in both AD-MCI and AD-D [44]. Results from the current study further and strongly support the involvement of the mesocorticolimbic pathway in AD, with loss of DAT activity and molecular connectivity in its major targets. Specifically, we observed loss of DAT activity in the ventral striatum and hippocampus, and in the middle cingulate gyrus, receiving dopaminergic input from the ventral and dorsal portions of VTA, respectively [45] (Fig. 5). Widespread molecular connectivity alterations affect both subcortical and cortical targets, indicating the involvement of the major VTA projections, including the meso-limbic, meso-cortical, meso-hippocampal, and meso-amygdala routes (cf. [33]) (see Fig. 5). The finding of a prominent involvement of the mesocorticolimbic system in AD is particularly relevant, as some of the VTA targets, and most prominently the ventral striatum, represent crucial hubs in the complex feed-forward organization of the dopaminergic circuitry. Notably, the ventral striatum, while receiving inputs from a relatively small population of neurons in the VTA, sends back widespread projections to both the VTA and the SN, hence being able to affect dopaminergic activity in widespread portions of the dorsal striatum, particularly in its associative regions (cf. [42]). This evidence raises the question of whether our findings of altered dopaminergic activity in the caudate nucleus might be consequential to the finding of altered dopaminergic function in the ventral striatum.

While our findings show a prominent involvement of the mesocorticolimbic system and a very limited involvement of the nigrostriatal targets, namely the caudatum, the pathogenic mechanisms underlying dopaminergic vulnerability in AD remain unknown. Some authors have suggested that the peculiar physiology of midbrain ventrategmental dopaminergic neurons, per se, might render those dopaminergic neurons particularly vulnerable to amyloid pathology in AD [36].

A link between dopaminergic dysfunction and tau pathology has also been hypothesized (cf. [39]). In this direction, a recent post-mortem study on human brain tissue reported an association between tau pathology and dysregulation of genetic pathways linked to dopaminergic neurotransmission [46]. Notably, the earliest tau site in AD, namely the locus coeruleus (LC), sends substantial noradrenergic afferents to midbrain dopaminergic nuclei, providing trophic support to both the VTA and the medial portions of SN pars compacta [47]. While the hypothesis that tau pathology in the LC is at the basis of the dopaminergic deficits observed in AD remains speculative, it is worth noting that the topography of dopaminergic alteration detected in our cohorts, specifically encompassing projections from the VTA and the medial portions of the SN pars compacta (Fig. 5), closely matches that of the noradrenergic projections from the LC to the midbrain dopaminergic nuclei [48]. Alternatively, it can also be hypothesized a direct effect of LC on the dopaminergic function of mesocorticolimbic targets, such as the hippocampus, that receive substantial dopaminergic input directly from LC’s tyrosine hydroxylase-immunoreactive neurons [49].

### Limitations

This study has some limitations. While all subjects (MCI and AD) were within the AD spectrum and underwent amyloid biomarker assessment [29], we do not have post-mortem pathology confirmation. Thus, we cannot exclude that some of them had additional co-pathologies. Moreove﻿r, the same diagnostic panel of biomarkers was not applied in each included subject, as the participating centers used either CSF or different imaging (amyloid-PET or FDG-PET, MRI) in the diagnostic algorithm. Thus, we cannot provide an additional characterization of the included cohorts according to the 2018 criteria [29]. We lack information on the prevalence of AD pathophysiology in our cohort of HC. However, we excluded the presence of cognitive impairment by means of the neurological examination and clinical follow-up.

As this represents an international multicentric and retrospective study, neuropsychological data were obtained with different tests and in different languages. This prevents us from evaluating the association between clinical measures and imaging data, which should be addressed by future studies.

We also acknowledge that while [123I]FP-CIT primarily measures DAT density, bindings to the serotonergic transporter should be considered in the extra-striatal regions. However, in vitro experiments have shown that [123I]FP-CIT has a high affinity for the DAT (low nanomolar range), a moderate affinity for the serotonin transporter (SERT), and a negligible affinity for the norepinephrine transporter [50, 51]. It is well accepted that striatal [123I]FP-CIT binding predominantly reflects binding to the DAT [52, 53]. Notably, DAT and SERT display a non-overlapping distribution in the brain, with higher specificity in basal ganglia for DAT [54]. Instead, approximately 70% of binding in the thalamus is from SERT (Koch et al., 2014). Since we limited our analysis exclusively to regions belonging to the nigrostriatal and mesocorticolimbic structures and pathways, very rich in dopamine transporters [34], we believe the interpretation of our findings in terms of alterations in DAT density is solid.

Last, we acknowledge the absence of correction for partial volume effects might represent a limit, also considering age- or AD-related functional changes; however, the combined use of (i) anatomical and functional probabilistic atlases for ROI segmentation, (ii) the strategy of picking only the center of each volume, and (iii) use of non-smoothed SPECT images should minimize the influence of partial volume effects. This strategy represents in addition the only solution that can be applied without the need of structural MRI, not available for all our subjects.

## Conclusions

Our study provides biological in vivo evidence for a significant derangement of the meso-limbic dopaminergic system in AD, already plateauing in the prodromal stages. Our data, strongly supported by statistical analyses of both in vivo dopaminergic binding density and molecular connectivity, point to different degrees of vulnerability of the dopaminergic afferents from specific dopaminergic nuclei. The mechanisms underlying the vulnerability of the dopaminergic circuitry in AD and the relationship with specific clinical aspects as well as the contribution of different genotypes need to be investigated with integrated multidisciplinary approaches, to foster results that might be relevant for new perspectives in early diagnosis, symptomatic treatment, and drug discovery in AD.

## Supplementary Information

**Additional file 1: Table A.1.** Results of the regional [123I]FP-CIT SBR in the major subcortical/cortical dopaminergic targets in the study groups.

## Data Availability

The datasets used in this study are available from the corresponding author upon reasonable request.

## Abbreviations

AD: Alzheimer’s disease
AD-D: Alzheimer’s disease dementia
MCI: Mild cognitive impairment
VTA: Ventral tegmental area
SN: Substantia nigra
DAT: Dopamine transporter

## References

[1] Reisine TD, Yamamura HI, Bird ED, Spokes E, Enna SJ (1978). Pre- and postsynaptic neurochemical alterations in Alzheimer’s disease. Brain Res..

[2] Joyce JN, Smutzer G, Whitty CJ, Myers A, Bannon MJ (1997). Differential modification of dopamine transporter and tyrosine hydroxylase mRNAs in midbrain of subjects with Parkinson’s, Alzheimer’s with parkinsonism, and Alzheimer’s disease. Mov Disord.

[3] Gibb WR, Mountjoy CQ, Mann DM, Lees AJ (1989). The substantia nigra and ventral tegmental area in Alzheimer’s disease and Down’s syndrome. J Neurol Neurosurg Psychiatry.

[4] Mann DMA, Yates PO, Marcyniuk B (1984). Monoaminergic neurotransmitter systems in presenile Alzheimer’s disease and in senile dementia of Alzheimer type. Clin Neuropathol..

[5] Rinne JO, SÄkö E, PaljÄrvi L, MölsÄ PK, Rinne UK (1986). Brain dopamine D-1 receptors in senile dementia. J Neurol Sci.

[6] Attems J, Quass M, Jellinger KA (2007). Tau and α-synuclein brainstem pathology in Alzheimer disease: relation with extrapyramidal signs. Acta Neuropathol..

[7] Gottfries CG, Adolfsson R, Aquilonius SM, Carlsson A, Eckernas SA, Nordberg A (1983). Biochemical changes in dementia disorders of Alzheimer type (AD/SDAT). Neurobiol Aging..

[8] Aral H, Kosaka K, Iizuka R (1984). Changes of biogenic amines and their metabolites in postmortem brains from patients with Alzheimer-type dementia. J Neurochem..

[9] Cross AJ, Crow TJ, Johnson JA, Joseph MH, Perry EK, Perry RH (1983). Monoamine metabolism in senile dementia of Alzheimer type. J Neurol Sci..

[10] Murray AM, Weihmueller FB, Marshall JF, Hurtig HI, Gottleib GL, Joyce JN (1995). Damage to dopamine systems differs between Parkinson’s disease and Alzheimer’s disease with parkinsonism. Ann Neurol Off J Am Neurol Assoc Child Neurol Soc.

[11] Storga D, Vrecko K, Birkmayer JGD, Reibnegger G (1996). Monoaminergic neurotransmitters, their precursors and metabolites in brains of Alzheimer patients. Neurosci Lett.

[12] Sweet RA, Hamilton RL, Healy MT, Wisniewski SR, Henteleff R, Pollock BG (2001). Alterations of striatal dopamine receptor binding in Alzheimer disease are associated with Lewy body pathology and antemortem psychosis. Arch Neurol..

[13] Seeman P, Bzowej N, Guan H, Bergeron C, Reynolds G, Bird E (1987). Human brain D1 and D2 dopamine receptors in schizophrenia, Alzheimer’s, Parkinson’s, and Huntington’s diseases. Neuropsychopharmacology..

[14] Kumar U, Patel SC (2007). Immunohistochemical localization of dopamine receptor subtypes (D1R-D5R) in Alzheimer’s disease brain. Brain Res..

[15] Cross AJ, Crow TJ, Ferrier IN, Johnson JA, Markakis D. Striatal dopamine receptors in Alzheimer-type dementia. Neurosci Lett. 1984;52(1–2):1–6. 10.1016/0304-3940(84)90341-052(1-2):1-6. 10.1016/0304-3940(84)90341-0 6241300

[16] Rinne JO, SÄkö E, PaljÄrvi L, MölsÄ PK, Rinne UK (1986). Brain dopamine D-2 receptors in senile dementia. J Neural Transm..

[17] Cortés R, Probst A, Palacios JM (1988). Decreased densities of dopamine D1 receptors in the putamen and hippocampus in senile dementia of the Alzheimer type. Brain Res..

[18] Joyce JN, Kaeger C, Ryoo H, Goldsmith S (1993). Dopamine D2 receptors in the hippocampus and amygdala in Alzheimer’s disease. Neurosci Lett..

[19] Rinne JO, Sahlberg N, Ruottinen H, Någren K, Lehikoinen P (1998). Striatal uptake of the dopamine reuptake ligand [11C]β-CFT is reduced in Alzheimer’s disease assessed by positron emission tomography. Neurology..

[20] Pizzolato G, Chierichetti F, Fabbri M, Cagnin A, Dam M, Ferlin G (1996). Reduced striatal dopamine receptors in Alzheimer’s disease: single photon emission tomography study with the D2 tracer [123I]-IBZM. Neurology..

[21] Reeves S, Brown R, Howard R, Grasby P (2009). Increased striatal dopamine (D2/D3) receptor availability and delusions in Alzheimer disease. Neurology..

[22] Meguro K, Itoh M, Yanai K, Takase K, Yamaguchi S, Ido T (1997). Psychiatric wandering behaviour in dementia patients correlated with increased striatal dopamine D2 receptor as shown by [11C]YM-09151-2 and positron emission tomography. Eur J Neurol..

[23] Kemppainen N, Ruottinen H, Någren K, Rinne JO (2000). PET shows that striatal dopamine D1 and D2 receptors are differentially affected in AD. Neurology..

[24] Kemppainen N, Laine M, Laakso MP, Kaasinen V, Någren K, Vahlberg T (2003). Hippocampal dopamine D2 receptors correlate with memory functions in Alzheimer’s disease. Eur J Neurosci..

[25] Sala A, Perani D (2019). Brain molecular connectivity in neurodegenerative diseases: recent advances and new perspectives using positron emission tomography. Front Neurosci..

[26] McKhann GM, Knopman DS, Chertkow H, Hyman BT, Jack CR, Kawas CH (2011). The diagnosis of dementia due to Alzheimer’s disease: recommendations from the National Institute on Aging-Alzheimer’s Association workgroups on diagnostic guidelines for Alzheimer’s disease. Alzheimers Dement.

[27] Albert MS, DeKosky ST, Dickson D, Dubois B, Feldman HH, Fox NC (2011). The diagnosis of mild cognitive impairment due to Alzheimer’s disease: recommendations from the National Institute on Aging-Alzheimer’s Association workgroups on diagnostic guidelines for Alzheimer’s disease. Alzheimers Dement..

[28] Darcourt J, Booij J, Tatsch K, Varrone A, Vander Borght T, Kapucu ÖL (2010). EANM procedure guidelines for brain neurotransmission SPECT using 123 I-labelled dopamine transporter ligands, version 2. Eur J Nucl Med Mol Imaging.

[29] Jack CR, Bennett DA, Blennow K, Carrillo MC, Dunn B, Haeberlein SB (2018). NIA-AA research framework: toward a biological definition of Alzheimer’s disease. Alzheimers Dement.

[30] Nicastro N, Garibotto V, Poncet A, Badoud S, Burkhard PR (2016). Establishing on-site reference values for 123 I-FP-CIT SPECT (DaTscan®) using a cohort of individuals with non-degenerative conditions. Mol imaging Biol.

[31] Pilotto A, Di Cola FS, Premi E, Grasso R, Turrone R, Gipponi S (2019). Extrastriatal dopaminergic and serotonergic pathways in Parkinson’s disease and in dementia with Lewy bodies: a 123 I-FP-CIT SPECT study. Eur J Nucl Med Mol Imaging.

[32] Gómez FJG, Huertas I, Ramírez JAL, Solís DG (2018). Elaboración de una plantilla de SPM para la normalización de imágenes de PET con 18F-DOPA. Imagen Diagnóstica..

[33] Iaccarino L, Sala A, Caminiti SP, Presotto L, Perani D (2020). In vivo MRI structural and PET metabolic connectivity study of dopamine pathways in Alzheimer’s disease. J Alzheimers Dis..

[34] Ciliax BJ, Drash GW, Staley JK, Haber S, Mobley CJ, Miller GW (1999). Immunocytochemical localization of the dopamine transporter in human brain. J Comp Neurol..

[35] Xia M, Wang J, He Y (2013). BrainNet Viewer: a network visualization tool for human brain connectomics. PLoS One..

[36] Nobili A, Latagliata EC, Viscomi MT, Cavallucci V, Cutuli D, Giacovazzo G (2017). Dopamine neuronal loss contributes to memory and reward dysfunction in a model of Alzheimer’s disease. Nat Commun..

[37] Cordella A, Krashia P, Nobili A, Pignataro A, La Barbera L, Viscomi MT (2018). Dopamine loss alters the hippocampus-nucleus accumbens synaptic transmission in the Tg2576 mouse model of Alzheimer’s disease. Neurobiol Dis.

[38] Tyrrell PJ, Sawle GV, Ibanez V, Bloomfield PM, Leenders KL, Frackowiak RS (1990). Clinical and positron emission tomographic studies in the ‘extrapyramidal syndrome’ of dementia of the Alzheimer type. Arch Neurol..

[39] De Marco M, Venneri A (2018). Volume and connectivity of the ventral tegmental area are linked to neurocognitive signatures of Alzheimer’s disease in humans. J Alzheimers Dis.

[40] Serra L, D’Amelio M, Di Domenico C, Dipasquale O, Marra C, Mercuri NB (2018). In vivo mapping of brainstem nuclei functional connectivity disruption in Alzheimer’s disease. Neurobiol Aging.

[41] Mann DMA, Lincoln J, Yates PO, Stamp JE, Toper S (1980). Changes in the monoamine containing neurones of the human CNS in senile dementia. Br J Psychiatry..

[42] Haber SN, Knutson B (2010). The reward circuit: linking primate anatomy and human imaging. Neuropsychopharmacology.

[43] Wise EA, Rosenberg PB, Lyketsos CG, Leoutsakos JM (2019). Time course of neuropsychiatric symptoms and cognitive diagnosis in National Alzheimer’s Coordinating Centers volunteers. Alzheimers Dement Diagn Assess Dis Monit..

[44] Lyketsos CG, Carrillo MC, Ryan JM, Khachaturian AS, Trzepacz P, Amatniek J (2011). Neuropsychiatric symptoms in Alzheimer’s disease.

[45] Swanson LW (1982). The projections of the ventral tegmental area and adjacent regions: a combined fluorescent retrograde tracer and immunofluorescence study in the rat. Brain Res Bull..

[46] Tiernan CT, Ginsberg SD, He B, Ward SM, Guillozet-Bongaarts AL, Kanaan NM (2018). Pretangle pathology within cholinergic nucleus basalis neurons coincides with neurotrophic and neurotransmitter receptor gene dysregulation during the progression of Alzheimer’s disease. Neurobiol Dis..

[47] Hassani OK, Rymar VV, Nguyen KQ, Huo L, Cloutier JF, Miller FD (2020). The noradrenergic system is necessary for survival of vulnerable midbrain dopaminergic neurons: implications for development and Parkinson’s disease. Neurobiol Aging..

[48] Kelly SC, He B, Perez SE, Ginsberg SD, Mufson EJ, Counts SE (2017). Locus coeruleus cellular and molecular pathology during the progression of Alzheimer’s disease. Acta Neuropathol Commun..

[49] Takeuchi T, Duszkiewicz AJ, Sonneborn A, Spooner PA, Yamasaki M, Watanabe M (2016). Locus coeruleus and dopaminergic consolidation of everyday memory. Nature.

[50] Abi-Dargham A, Gandelman MS, DeErausquin GA, Zea-Ponce Y (1996). SPECT imaging of dopamine transporters in human brain with iodine-123-fluoroalkyl analogs of beta-CIT. J Nucl Med.

[51] Scheffel U, Lever JR, Abraham P, Parham KR, Mathews WB, Kopajtic T (1997). N-substituted phenyltropanes as in vivo binding ligands for rapid imaging studies of the dopamine transporter. Synapse.

[52] Booij J, Tissingh G, Boer GJ, Speelman JD, Stoof JC, Janssen AG (1997). [123I] FP-CIT SPECT shows a pronounced decline of striatal dopamine transporter labelling in early and advanced Parkinson’s disease. J Neurol Neurosurg Psychiatry.

[53] Andringa G, Drukarch B, Bol JGJM, de Bruin K, Sorman K, Habraken JBA (2005). Pinhole SPECT imaging of dopamine transporters correlates with dopamine transporter immunohistochemical analysis in the MPTP mouse model of Parkinson’s disease. Neuroimage.

[54] Koch W, Unterrainer M, Xiong G, Bartenstein P, Diemling M, Varrone A, Dickson JC, Tossici-Bolt L, Sera T, Asenbaum S, Booij J, Kapucu OL, Kluge A, Ziebell M, Darcourt J, Nobili F, Pagani M, Hesse S, Vander Borght T, Van Laere K, Tatsch K, la Fougère C. Extrastriatal binding of [¹²³I]FP-CIT in the thalamus and pons: gender and age dependencies assessed in a European multicentre database of healthy controls. Eur J Nucl Med Mol Imaging. 2014;41(10):1938–46. 10.1007/s00259-014-2785-8. 10.1007/s00259-014-2785-8 24806112

