# Supplementary material for: In vivo human molecular neuroimaging of dopaminergic vulnerability along the Alzheimer’s disease phases
Source: Alzheimers Res Ther. 2021 Nov 12;13:187. doi: 10.1186/s13195-021-00925-1 (PMC8588696; doi:10.1186/s13195-021-00925-1)
Supplement: Supplementary file 1 — Additional file 1: Table A.1. Results of the regional [123I]FP-CIT SBR in the major subcortical/cortical dopaminergic targets in the study groups. [file 13195_2021_925_MOESM1_ESM.docx]

**Table A.1. Results on the regional [123I]FP-CIT SBR in the major subcortical/cortical dopaminergic targets in the study groups**

|  | **AD-D** | **AD-MCI** | **HC** | **F- value** | **p-value** |
| --- | --- | --- | --- | --- | --- |
| ***Mesocorticolimbic targets*** | | | | |  |
| \| **Left Ventral Striatum, mean [SE]** \| \| --- \| | 2.19 [0.09] | 2.28[0.11] | 2.68[0.05] | 13.82 | <0.001* |
| **Right Ventral Striatum, mean [SE]** | 2.27 [0.09] | 2.29[0.11] | 2.70[0.05] | 11.98 | <0.001* |
| **Left Amygdala, mean [SE]** | 0.27[0.06] | 0.26[0.07] | 0.35[0.03] | 1.17 | 1.00 |
| **Right Amygdala, mean [SE]** | 0.30[0.06] | 0.28[0.07] | 0.37[0.03] | 1.01 | 1.00 |
| **Left Hippocampus, mean [SE]** | 0.11[0.03] | 0.15[0.04] | 0.24[0.02] | 6.69 | <0.05† |
| **Right Hippocampus, mean [SE]** | 0.17[0.04] | 0.16[0.05] | 0.30[0.02] | 6.72 | <0.05* |
| **Left Anterior Cingulate Cortex, mean [SE]** | 0[0.03] | 0.07[0.04] | 0.11[0.02] | 5.78 | 0.057 |
| **Right Anterior Cingulate Cortex, mean [SE]** | 0.03[0.03] | 0.03[0.04] | 0.08[0.02] | 1.00 | 1.00 |
| **Left Middle Cingulate Cortex, mean [SE]** | 0.06[0.03] | 0.07[0.04] | 0.15[0.02] | 4.29 | 0.227 |
| **Right Middle Cingulate Cortex, mean [SE]** | 0.02[0.03] | 0.06[0.04] | 0.14[0.02] | 7.67 | <0.05† |
| ***Nigrostriatal targets*** | | | | |  |
| **Left Dorsal Caudate Nucleus, mean [SE]** | 1.63[0.11] | 1.93[0.14] | 2.41[0.06] | 21.49 | <0.001* |
| **Right Dorsal Caudate Nucleus, mean [SE]** | 1.44[0.10] | 1.76[0.13] | 2.13[0.06] | 17.61 | <0.001* |
| **Left Dorsal Putamen, mean [SE]** | 2.78[0.11] | 2.78[0.14] | 3.12[0.06] | 4.96 | 0.122 |
| **Right Dorsal Putamen, mean [SE]** | 2.84[0.11] | 2.74[0.14] | 3.17[0.06] | \| 5.52 \| \| --- \| | 0.073 |

*MANCOVA results are corrected for age, gender and reconstruction method.*

*Bonferroni-corrected p-values for multiple comparisons are shown.*

*Significant differences at post-hoc analysis, deemed significant at p<0.05, with Bonferroni-correction for multiple comparisons:*

* AD-D vs. HC and AD-MCI vs. HC

† AD-D vs. HC

*Abbreviations: AD-D=Alzheimer’s disease dementia; HC= healthy controls; AD-MCI= MCI due to Alzheimer’s disease; SE= standard error; SBR=specific binding ratio.*
